# The Organization and Diseases of the Teeth

**Published:** 1891-06

**Authors:** E. G. Tucker


### THE ORGANIZATION AND DISEASES OF THE TEETH.[1]

BY E. G. TUCKER, M.D.

MR. PRESIDENT AND FELLOWS OF THE ACADEMY,—As an *important branch*, I have selected that which relates to the organization of the teeth and a few remarks on the technical phrases of the teeth and diseases.

In attempting to explain the organization of the teeth, I shall not fatigue you with long and tedious recitals, or attempt a refutation of those propositions which neither result in a clear conviction of truth or useful knowledge, by doubting the very existence of my person and of others around me, until logically proved to exist.

That no reasoning of mine or of the ancients will be necessary to convince you, without the aid

> "Of ancient critics
> Although profoundly skilled in analytics,"

I find it difficult to summarize the many opinions advanced. Those who are acquainted with the writings of Fauchard, Monroe, Audibran, Robert Blake, Duval, Fox, Fitch, and Thomas Bell, or know the opinions of intelligent practitioners, doubtless think the question so well settled as to render further argument unnecessary.

The famous Mr. Lawrence, of London, published in the *Medical Gazette* of June, 1830, his opinions, and attributes all diseases of the teeth to a "chemical actiŏn." To say nothing of the absurdity of calling diseases a chemical action, I would ask, How then can these affections be inherited or constitutional? That many are so is not only a popular belief, but a truth to be learned by a very little observation. We see children as often resembling their parents in *dental* as in pulmonary or in any other peculiarity, complexion, etc.

The expression "the teeth of all our family go just so" is not only an assertion which a dentist has almost daily to hear, but one which *facts* compel him to give his assent.

Numberless facts on this point might be collected, but proof is not needed. Every candid observer can convince himself of the truth in one week, if such proof be necessary.

In many cases the application of cold or hot substances, and more

[1] Read before the American Academy of Dental Science, Boston, February 4, 1891.

especially of sweets or acids to parts denuded, occasions sensations so disagreeable as to render it difficult to convince the patient that the nerve or pulp is not exposed.

Teeth possess this irritability at times and at all periods of life; differing, of course, in degree like all other irritability or fretfulness in different individuals. The enamel is devoid of sensibility, but there is no other part of these organs which does not possess it, as bones do. Now what is it that is thus transmitted from one generation to another? Is it a liability to a chemical action, or are the teeth, like the lungs and other parts, rendered liable to or exempt from disease?

The late Dr. Harwood, of Boston, had a variety of specimens of diseased roots, and no one who examines them would hesitate to ascribe the peculiar appearance they present to genuine exostosis, not only the surface, but the substance of the superadded parts is of a very hard and dense texture, of a yellowish hue, and before being dried was semi-transparent. The roughness presents a striking contrast to healthy roots. Where does this matter come from, and how comes it to be so firmly united with and forming, as it were, a part of the root?

If it were a mere deposit, it would be easily separated from the original bone. It seems to me that the occurrence of this disease proves the existence of vessels in whatever part it may happen, and we might believe that the phenomena presents a morbid growth of the parts.

Chemistry has done wonders, but I am hardly prepared to believe that exostosis is one of its products.

I have seen teeth that have been buried more than one hundred and fifty years, but they presented none of the appearances just described. Nothing in fact does resemble a tooth thus affected but necrosed bone, and if not removed by art, nature accomplishes the thing herself.

These unorganized *pegs* not only manage to imitate diseases but counterfeit resemblance to them remarkably well. When by accident or design a portion of the enamel is removed, the exposed bone becomes irritable, but with proper treatment the tenderness in most cases disappears. Where this has happened, and the bone continues healthy, it is found, on close examination, that it has become much harder than ordinary bone or dentine, and that the surface has assumed a peculiar bright, glossy appearance; such facts have, no doubt, given rise to the erroneous idea that a new enamel is sometimes formed. Now, here is not only a modification of sensibility, but a change of structure which must depend upon vitality and organization as much as dental gangrene.

The diseases of the teeth have not received that attention desired heretofore, except when felt; then came the cold iron, an evil nearly equal to the prior one. The work of destruction is now and then interrupted by some internal power, and the organ remains for years.

I cannot pass this point without making a short digression, for which I hope to be pardoned. The vulgar phrase "rotten teeth" is not merely an indecent one, but it conveys to the uninformed mind an utter falsehood, and, I fear, is not always used with a proper understanding by those who should know that the teeth are incapable of such a process as rotting, any more than the flesh of a healthy man. That process which is termed "rotting" of teeth, or, sometimes, for decency, "decaying," ends totally at death, and never is or can be continued in the grave.

Extract a tooth and you immediately stop its so-called "rotting," nor can you by any means make it go on except under artificially produced conditions similar to those that caused the decay in the mouth.

In most cases, if people knew the nature of the evil, they would attend more to the prevention, if not to the cure.

Cleanliness would at least be attended to, and I honestly believe that half the teeth that are lost might be saved.

People may have foul mouths full of corrupt substances, and thus inflammation and decay may be produced in their teeth; but let them be told and made to understand that their teeth can take on no such process until after they are softened and prepared by disease, which is in most cases easily prevented or cured.

Physicians, of all men, should be careful how they use this ill-chosen phrase. It has a technical and generally understood signification; its legitimate application is to *dead* matter. If, therefore, you tell a man that his teeth are rotting, you virtually tell him that they are in a hopeless condition, and he acts accordingly in nine cases out of ten.

The late Dr. Flagg succeeded in coloring the whole tooth by mixing madder in the animal's food, and also Dr. Fitch, of Philadelphia, states that he had several specimens of extracted teeth of a plethoric woman, aged twenty-seven years, who died of a violent inflammatory fever, and the teeth were completely injected with red blood.

We see the roots covered with periosteum like other bones, and a large number of vessels enter this membrane of such magnitude as to bleed profusely when torn asunder.

Is it philosophical to suppose that *arteries* and *veins* are sent thither for the mere purpose of coming back again? or that the nerves that accompany them have no other end to answer than that of subjecting us to the vexation and madness of toothache?

We know that teeth can be transplanted from one jaw to another; which, though injudicious, has in some instances been successful.

There are few organs which are more important in the influence which they exert upon the general health than the teeth, and there are few diseases so much neglected and which receive so little attention from the hands of the medical practitioners. Lately, however, they seem to have greatly changed, and their reluctance heretofore manifested is giving place to more rational views, and we find now many of them ardently devoting themselves to what was once considered beneath the dignity of their calling.

A grave impression, somewhat prevalent, is this: that the diseases of the teeth are a natural consequence, a process of nature which none are exempt from, and that it is not within the power of the dentist to check its ravages.

An interrogatory very often sounds in our ears, namely, "Why, if filling the teeth is so essential, how is it that we find so many failures?" The solution of the query is, that such persons or services as would insure success have not been employed for the sake of economy, instead of which an empiric has been called upon to do the work.

There are many persons in the world who are too "*penny-wise*" to make use of any other wisdom, judging the dental science to be a mere mechanical one, and, to use the words of another, "this is the rock on which they split."

However natural it may be for us all to indulge in a partiality for those subjects which have engaged much of our time and attention, yet every one must agree that the advantages of this art, aside from its effects upon the general health, are sufficient to excite our laudable and generous efforts in diffusing its principles in the community. The vast importance of preserving the organs of mastication and articulation is apparent to every one, as an indispensable requisite to sound bodily health, for there are many affections which arise solely from the diseases of the teeth, such as dyspepsia, tic douloureux, etc. Still, there are no operations in surgery which, when performed as they should be, are more certainly successful in their results.

